# Student Leadership Development in a Gen‐AI Higher Education Landscape: Four Imperatives for the Future

**DOI:** 10.1002/yd.70062

**Published:** 2026-05-21

**Authors:** Kris Acheson

**Affiliations:** ^1^ Leadership and Professional Development Purdue University West Layafette Indiana USA

## Abstract

Artificial intelligence (AI) is transforming higher education, yet student leadership programs have failed to engage this transformation. This article examines four imperatives for integrating AI into leadership education: AI as pedagogy, AI literacy as leadership competency, student agency in institutional AI transformation, and AI‐enhanced assessment of leadership development. I argue leadership educators have opportunities and responsibilities to lead, not only react to, higher education's AI engagement. A framework spanning educator actions, programmatic redesign, institutional transformation, and field‐level initiatives is offered as a response. By embracing these imperatives, leadership development can responsibly integrate AI while preparing students for future AI‐driven challenges.

## 1 Leadership Education at a Critical Juncture

Artificial intelligence (AI) is no longer an emerging technology on higher education's horizon. It has arrived, and its implications for teaching, learning, and student development are still coming into focus. Leadership education — the intentional, systematic integration of leadership development into both curricular and co‐curricular experiences in higher education (Dugan 2011; Komives and Sowcik 2020) — has an unusual opportunity and an unusual obligation in this moment. This article examines what that opportunity demands: how leadership educators can and should engage with AI as a pedagogical tool, as a subject of student learning, as a site of institutional transformation, and as a resource for assessment. The convergence of AI and leadership development is already underway on campuses across the country, and the decisions institutions make now will shape the field for years to come.

In fall 2025, Purdue University became the first university in the United States to implement AI competency graduation requirements for all undergraduate students, including Learning with AI, Learning About AI, Researching AI, Using AI and Partnering in AI (Salai 2026). This institutional mandate by Purdue, Indiana's land grant university and a global leader in STEM education, signals a profound shift in how universities conceptualize essential literacies for the 21st century. Yet this requirement does more than simply add another checkbox to degree completion; it fundamentally raises questions about what it means to prepare students for citizenship, work, and life in an AI‐integrated world.

At Purdue, this AI competency requirement intersects meaningfully with the university's LEAD framework—a strengths‐based, competency‐driven approach to leadership development designed for all undergraduates (Purdue University n.d.). In an institution serving a highly internationalized student body with an explicitly future‐ready vision, the convergence of AI competency and leadership competency development represents both a local imperative and a bellwether for the broader field. What Purdue is beginning to grapple with institutionally reflects a broader reality: AI is reshaping the conditions for leadership development across higher education, whether institutions have engaged that fact deliberately or not.

Despite AI's rapid integration into higher education, leadership development scholarship and practice have been conspicuously slow to engage. Systematic reviews confirm that AI applications cluster in technical disciplines and administrative functions, leaving student affairs and leadership education largely untouched (Chan and Hu 2023; Liang et al. 2025; Zawacki‐Richter et al. 2019). Where the broader field has responded, the results have been uneven at best, and for leadership education specifically, the stakes of continued inaction are uniquely high.

Where higher education has responded to AI in scholarly literature, the responses are often fear‐driven prohibition, superficial engagement, or passive acceptance (Bond et al. 2024; Chiu 2024; Crompton & Burke, 2023). Many educators treat AI primarily as a threat to academic integrity rather than as a transformative force requiring thoughtful engagement (Bearman et al., 2023). Others bolt on cursory AI modules without reconceptualizing intended outcomes or teaching practices (Bocconi et al., 2024; Ren & Wu, 2025), while still others seem to be ignoring the issue entirely.

Leadership education faces a responsibility in this moment that extends beyond general calls for digital literacy or technological adaptation. Leadership development is fundamentally about preparing students to navigate complex, ambiguous challenges, make ethical decisions under uncertainty, and create positive change in communities and organizations (Day et al. 2014 precisely the capacities most needed in an AI‐integrated world. If leadership education cannot model thoughtful, ethical engagement with AI, it can hardly expect students to demonstrate such leadership themselves.

This article addresses that gap directly, offering a conceptual framework that gives leadership educators both a rationale and a roadmap for substantive AI integration. Four imperatives guide that framework:

1. Leveraging AI as a pedagogical tool to enhance leadership learning,
2. Developing AI literacy as a core leadership competency,
3. Positioning students as agents in institutional AI transformation, and
4. Utilizing AI to enhance assessment of leadership development.

These imperatives are not optional enhancements or peripheral considerations; they are essential to maintaining the relevance and rigor of leadership education in a rapidly changing landscape. The argument is quite simple: Ignoring these imperatives leaves students unprepared for the actual leadership challenges they will face in their university life and the working world beyond. Leadership educators can and must do better. We have an opportunity to lead higher education's engagement with AI, modeling how to integrate powerful new tools while maintaining pedagogical integrity, fostering critical thinking, and centering human connection and growth.

The following section examines each imperative through three lenses: the current state of practice, why meaningful engagement matters, and what effective integration looks like in leadership education contexts.

## 2 Imperatives for AI in Leadership Learning

### 2.1 Imperative 1: AI as a Pedagogical Tool

The current integration of AI in higher education has been dominated by defensive reactions rather than proactive pedagogical innovation. University faculty and staff have primarily responded to the rising use of generative AI as threats implemented ChatGPT bans, plagiarism detection efforts, and academic integrity policies (Lee et al. 2024; Liang et al. 2025). While these concerns about academic honesty are valid, this defensive posture has prevented educators from exploring how AI might enhance learning experiences. Even institutions that have moved beyond prohibition often engage with AI superficially, perhaps allowing students to use ChatGPT for brainstorming or adding a module on AI tools to existing coursework.

This surface‐level integration fails to leverage AI's deeper pedagogical possibilities or to model the sophisticated, critical engagement with AI that students will need as leaders. The result is a significant missed opportunity: AI tools capable of providing personalized feedback, scaffolding complex thinking, and enabling scaled practice opportunities remain underutilized. This gap between AI's potential and its current use is as true for leadership development as it is for higher education broadly.

Leadership learning possesses unique characteristics that make it particularly well‐suited to AI‐enhanced pedagogy. Unlike knowledge acquisition in many disciplines, leadership development based on transformative learning (Mezirow 1991; Guthrie and Jenkins 2018) and other models require extensive reflection, iterative practice, personalized feedback, and the ability to test approaches in lower‐stakes environments before applying them in consequential situations. These are precisely the areas where generative AI can provide substantial value.

Reflection is a fundamental process in experiential learning, serving as the mechanism through which experience is transformed into knowledge (Kolb 1984). In leadership education specifically, reflection functions as an essential pedagogical tool, the means by which students mine their experiences to develop leadership knowledge, skills, and self‐awareness (White & Guthrie, 2016). GenAI systems can enhance this process through guided prompting and feedback, asking probing questions that push students deeper into their thinking, identifying patterns in students' reflective writing over time, and offering personalized suggestions for areas to explore further (Al‐Zahrani 2024). This doesn't replace the irreplaceable value of human mentorship—rather, it allows educators to focus their limited time on the highest‐touch, most complex mentoring interactions while AI handles more routine guidance.

Similarly, AI can scaffold complex thinking and personalized learning in ways naturally aligned with strengths‐based development approaches. An AI system can learn a student's particular strengths profile and suggest leadership scenarios and development activities tailored to both leverage existing strengths and develop emerging capacities (Al‐Zahrani 2024). An apt example is Gallup's new AI‐driven debriefing tool that participants who take the CliftonStrengths assessment can use to reflect on their survey results and think through the implications of their strengths (Gallup n.d.). A new online self‐driven learning module Purdue has released to support student leadership development directs learners to this AI tool to leverage the CliftonStrengths formative assessment into career preparation and individualized developmental journey planning. This kind of mass customization was previously impossible without dramatically increasing educator workload, but AI enables personalized learning at scale.

Perhaps most importantly, AI can provide safe spaces for practicing risky social learning. Leadership inevitably involves difficult conversations—giving critical feedback, navigating conflict, addressing equity issues, or challenging organizational norms. Students need opportunities to practice these high‐stakes interactions before facing them in consequential settings. AI‐powered simulations can allow students to engage in these challenging scenarios, make mistakes, receive feedback, and try again without real‐world consequences. Research on simulation‐based learning demonstrates how, in contrast to in‐class role plays or real‐world interactions that carry relational and emotional consequences, AI can support experimentation and practice of relational leadership skills by providing a safe learning environment for potentially uncomfortable social interactions (Akdere et al. 2023).

Recent empirical evidence supports this potential: A randomized controlled trial found that a pedagogically designed AI tutor produced learning gains more than double those of in‐class active learning, while also reporting higher student engagement and motivation (Kestin et al. 2025). Although this experiment was conducted in a STEM setting, the findings suggest that, by thoughtfully integrating AI as a pedagogical tool, leadership education can model for undergraduate educators more broadly how to leverage AI capability while avoiding its pitfalls. Leadership educators can demonstrate what it means to use AI ethically, to foster rather than erode critical thinking through AI integration, and to maintain the humanity—the connection, meaningfulness, and transformative potential—of education even as we incorporate powerful new tools (Bond et al. 2024). This modeling function is itself an important contribution to higher education's AI transformation.

### 2.2 Imperative 2: AI Literacy as a Leadership Competency

A scan of prominent leadership competency frameworks reveals a notable absence: AI literacy rarely appears as an explicit leadership competency. The Student Leadership Competencies framework (Seemiller and Murray 2013), which has shaped leadership program design across hundreds of institutions, was developed before the current AI revolution and understandably does not address AI‐related capacities. More recent frameworks have also failed to integrate AI literacy systematically (Skalicky et al. 2020). Emerging efforts to address this gap exist. Yang and Jiménez‐Luque (2025) proposed an integrated model explicitly connecting AI‐enhanced learning with leadership development. The UNESCO (2024) AI Competency Framework for Students, while not a leadership framework per se, outlines competencies across four dimensions including ethics of AI and a human‐centered mindset with clear implications for how leadership education might conceptualize AI literacy. When AI‐related skills appear at all in undergraduate learning outcomes, they are typically treated as technical competencies—understanding how algorithms work or being able to use AI tools—rather than as leadership imperatives requiring ethical judgment, strategic thinking, and organizational decision‐making.

One institutional example illustrates what this integration could look like in practice. Purdue University's LEAD Competency Framework organizes student leadership development across four domains (i.e., Communication, Self‐Awareness, Collaboration, and Ways of Thinking) each progressing through four developmental levels. Mapping these existing competencies against the UNESCO (2024) AI Competency Framework for Students, which identifies 12 competencies across four dimensions (i.e., human‐centred mindset, ethics of AI, AI techniques and applications, and AI system design), reveals seven natural integration points where AI literacy extends rather than replaces established leadership capacities (Figure 1).

FIGURE 1Integrating AI Literacy into the Purdue LEAD Competency Framework: Seven Integration Points Across Two Levels. LEAD competency framework: Purdue University (n.d.). UNESCO AI Competency Framework: UNESCO (2024). Integration analysis: original.

**Figure:**
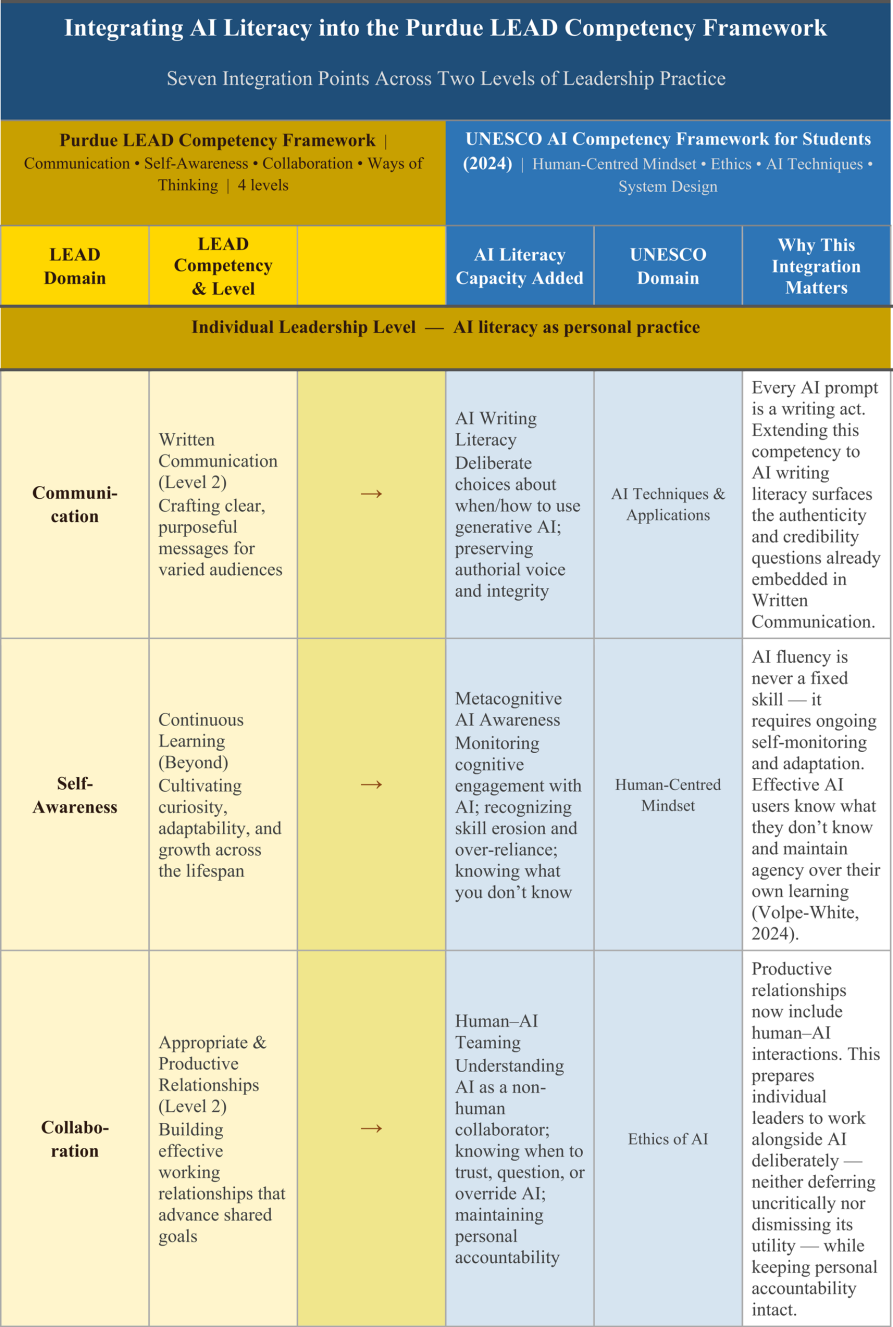

**Figure:**
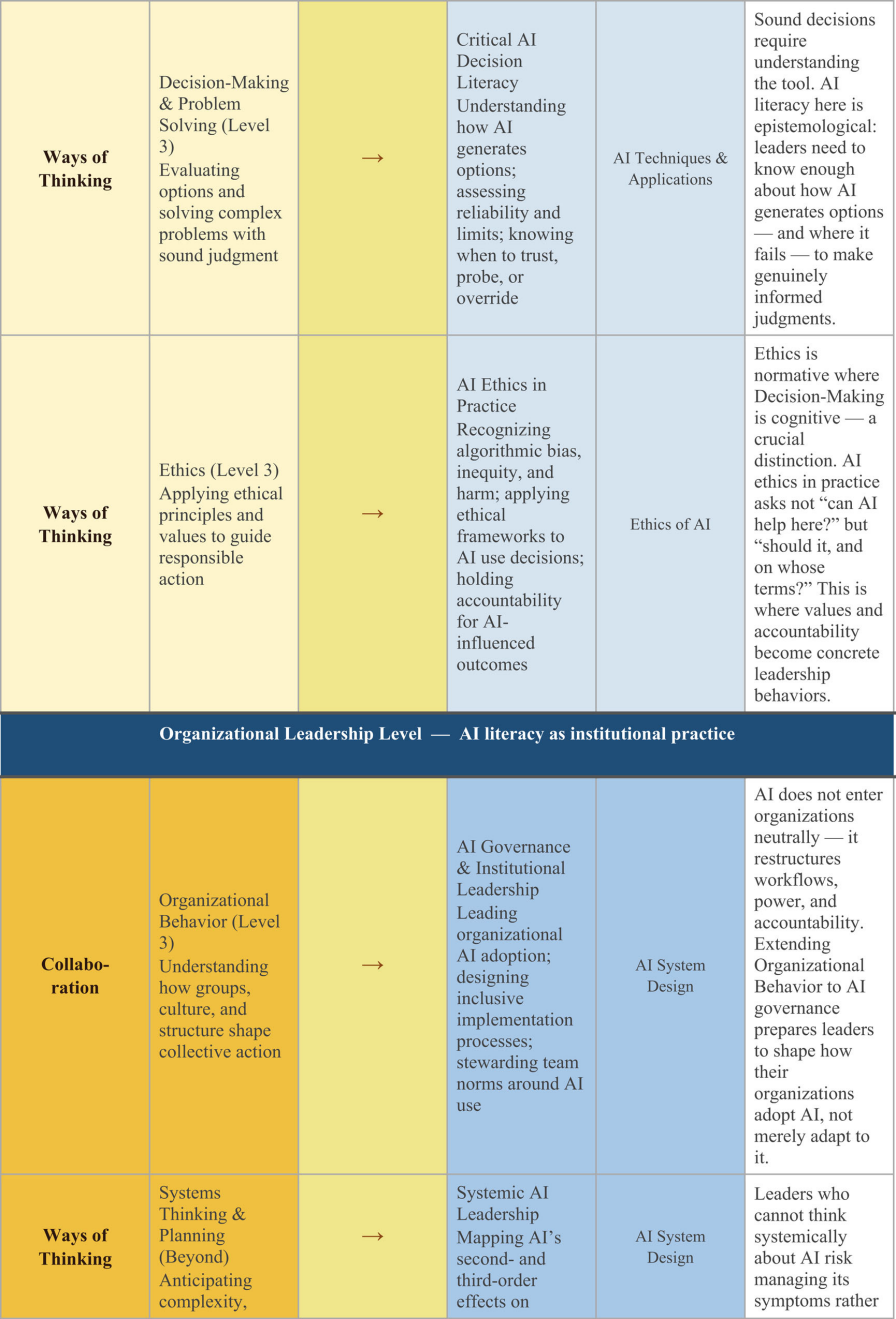

**Figure:**
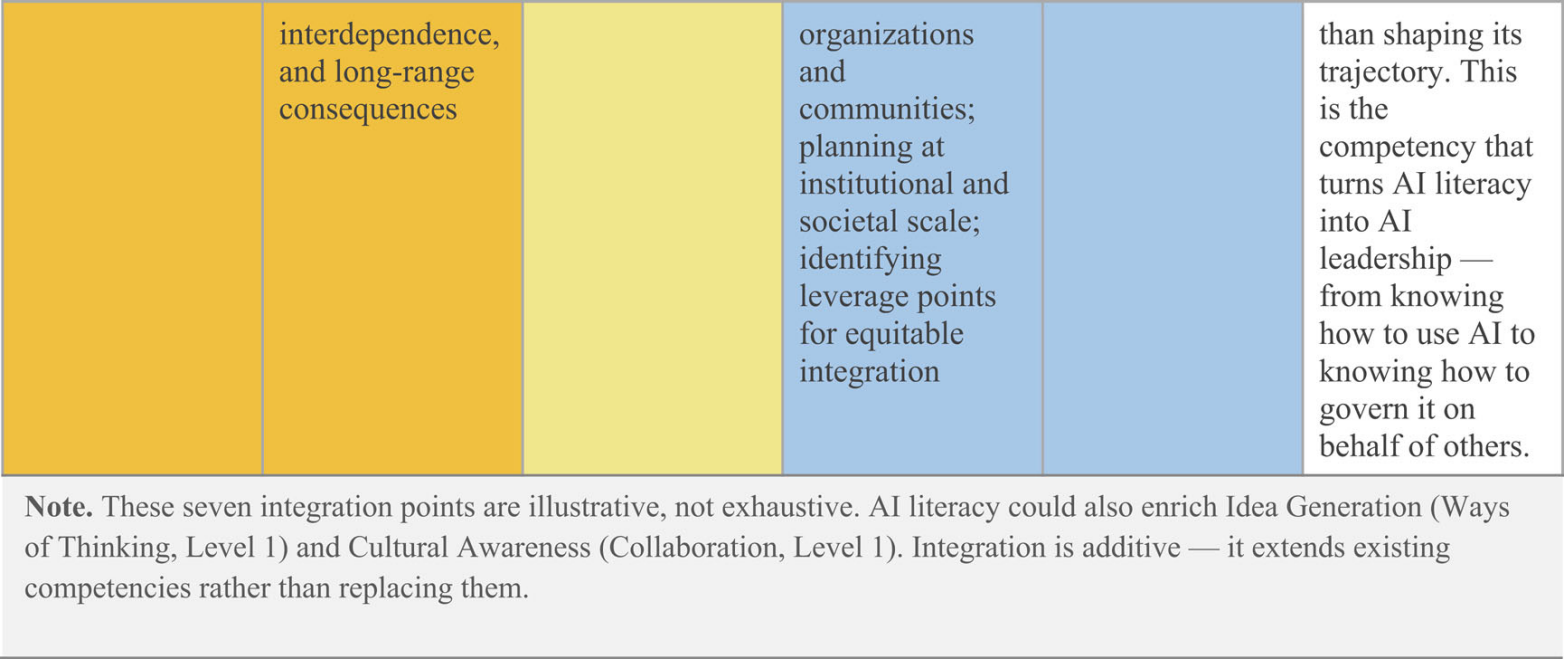

While this example demonstrates the feasibility of integrating AI into leadership competency frameworks, this approach has not yet been broadly adopted within mainstream leadership education. This absence matters because competency frameworks shape what leadership programs teach; how they design courses, trainings, and experiential learning opportunities; and what outcomes they assess. If AI literacy is not recognized as a leadership competency, it will continue to be relegated to IT departments or treated as a mere technical skill rather than as a fundamental contemporary leadership capacity.

Today's students will become tomorrow's leaders making consequential decisions about AI implementation in teams, organizations, and entire disciplines. These decisions will shape how AI affects human work, organizational culture, equity and access, and the very nature of human collaboration. Leaders making these decisions need to be well‐grounded in both the capacities and limitations of AI (Myszak and Filina‐Dawidowicz 2025). They need to understand what AI can and cannot do, how AI systems are trained and where they may contain biases, what data privacy and security issues arise from AI use, and how AI implementation affects different stakeholders.

Beyond technical understanding, the ethical implications of AI use demand leadership judgment. Leaders will need to decide when AI use is inappropriate in their contexts and what boundaries should be established to prevent harm. Recent controversies illustrate the high stakes of these leadership decisions: AI chatbots have provided harmful mental health advice including encouraging self‐harm (Frances 2026); deepfake technology has been used to create exploitative content, with 96% of deepfakes being nonconsensual and 99% of sexual deepfakes depicting women (Gural 2025); and AI hiring systems have perpetuated discrimination, with one study finding AI resume screeners preferred white‐associated names in 85% of cases (Wilson and Caliskan 2024). Technical knowledge alone is insufficient. Ethical reasoning, stakeholder analysis, and values‐based decision‐making are essential.

If humans are going to survive and thrive in an AI‐integrated world, leaders must continually and proactively engage in critical thinking about AI systems and their societal impacts (Madanchian et al. 2024). These issues include labor market disruptions as AI changes the nature of work, with research showing computer and mathematical occupations experiencing steep unemployment increases correlated with AI adoption (Ozkan and Sullivan 2025); skill erosion as we delegate cognitive tasks to machines (Salai 2026); the concentration of AI power in particular corporations and nations (UNESCO 2025); and the environmental costs (Ligozat et al. 2022) of training and running large AI models. Leaders across all sectors, not just technology sectors, will need to grapple with these implications.

Moreover, AI literacy has crucial intercultural dimensions (Al‐Zahrani 2024). AI systems trained primarily on Western, English‐language data may perpetuate cultural and racial biases — a pattern Yang and Jiménez‐Luque (2025) term cognitive imperialism, whereby AI embeds dominant epistemologies while marginalizing non‐Western and Indigenous leadership traditions. The global nature of AI development and deployment demands international cooperation in AI governance and regulation. Leaders working in internationalized contexts, which increasingly includes all contexts, must understand these cross‐cultural dynamics and be prepared to advocate for inclusive AI development and equitable AI access, including through support for transparent, open‐source approaches that better reflect diverse leadership traditions (Yang and Jiménez‐Luque 2025).

### 2.3 Imperative 3: Positioning Students as Agents in Institutional AI Transformation

In most institutional AI discussions, students are positioned as policy subjects rather than policy makers. University task forces on AI implementation typically comprise faculty, administrators, and perhaps industry representatives, while students—the primary stakeholders most affected by these policies—remain absent from decision‐making tables. When students are included, it is often in token ways: perhaps one student representative on a large committee, or students asked to provide feedback on already‐developed policies rather than participating in their creation.

This top‐down approach to institutional AI governance represents a profound missed opportunity for leadership development. At the very moment when institutions are making consequential decisions about how AI will shape teaching, learning, and campus life, students who could be developing real leadership capacity through authentic engagement with these issues are instead sidelined. The implicit message is troubling: Students are not yet ready to participate in decisions that directly affect them.

Generative AI is poised to dramatically disrupt higher education in the near future, demanding a paradigm shift in university programs and expectations of both educator and learner behaviors (Alotaibi 2024). The stakes of these decisions are not hypothetical; they will directly shape students' current educational experiences and their preparation for future careers. This makes AI governance a case of authentic leadership experience with genuine consequences (Skalicky et al. 2020). Rather than simulated leadership scenarios or low‐stakes practice, student engagement with institutional AI policy offers real leadership development with actual impact.

Students are not only stakeholders in these decisions; at times, their so‐called digital‐native perspective (Prensky 2001) means they bring genuine expertise and insight. Many current undergraduates have been using AI tools longer than their professors, experimenting with different applications, and discovering both possibilities and pitfalls through direct experience. While this experiential knowledge doesn't replace deeper technical understanding or pedagogical expertise, it is valuable and should inform institutional decision‐making. When institutions underutilize this student expertise, they make poorer decisions.

Furthermore, in contemporary higher education's complex landscapewhere students are increasingly viewed as consumers or clients (Bunce et al. 2017)—students may have more power than they realize to affect change in university AI policies and practices. Student advocacy has historically driven institutional change on issues from sustainability to mental health services to diversity initiatives (Cook‐Sather 2020). AI policy is similarly susceptible to organized student influence. Leadership development programs should help students recognize and exercise this agency productively. Creating meaningful roles for students in institutional AI transformation serves multiple purposes simultaneously, producing better institutional policies by including key stakeholders, providing students with high‐impact leadership development experiences, and modeling the kind of inclusive, participatory decision‐making that students should employ in their own future leadership roles.

### 2.4 Imperative 4: AI‐Enhanced Assessment of Leadership Development

Leadership development assessment currently faces a crisis of authenticity driven by AI's capabilities. When students use AI to generate reflective essays, complete self‐assessments, or produce evidence of learning, how can educators be completely confident their assessments capture student growth rather than AI performance? This crisis has prompted a defensive posture toward AI in assessment contexts (Swiecki et al. 2022). Educators have responded by adding AI detectors, shifting to high‐stakes in‐person assessments, or simply hoping that students will choose not to use AI tools when they could.

This defensive reaction, while understandable, misses a crucial opportunity. While worrying about whether AI threatens assessment authenticity, leadership educators have largely failed to explore how AI might enhance assessment practices. The same technologies that create authenticity concerns also offer unprecedented capabilities for analyzing student work, identifying patterns of growth, providing personalized feedback, and supporting the iterative, developmental process that effective leadership education requires. There is thus a simultaneous overreaction to AI's threats and underutilization of AI's potential in leadership assessment. This imbalance must be corrected if leadership development is to maintain rigorous, meaningful evaluation of student learning in an AI‐integrated landscape.

Leadership learning is fundamentally an iterative process requiring ongoing feedback and reflection at all stages of learning. Assessment in this context is not primarily about sorting students or assigning grades; it is about fostering student growth and documenting that growth over time (Seemiller and Murray 2013). Every assessment moment becomes both an opportunity to encourage deeper reflection by learners and a chance to capture evidence of development. This developmental, formative orientation to assessment makes leadership education particularly well‐suited to AI enhancement.

Competency‐based approaches to leadership development, which have become increasingly prevalent, require robust measurement systems (Day et al. 2014). These systems include both indirect assessment—capturing students' perspectives on their own growth, their confidence in various competencies, and their understanding of leadership concepts—and direct assessment of demonstrated skill levels through behavioral observation, performance tasks, and application of knowledge. Building and maintaining such comprehensive assessment systems is resource intensive. AI can make rigorous competency assessment more feasible on a large scale.

The pervasiveness of AI also means traditional assessment methods face new significant limitations. When students can use AI to complete reflective writing assignments or to respond to leadership scenarios, the validity of these measures become questionable. Are we assessing student leadership competencies or AI competencies? Rather than abandoning valuable assessment approaches or engaging in an arms race of AI detection, leadership educators should redesign assessment to account for AI's existence while leveraging AI's capabilities to enhance measurement.

Rigorous and systematic assessment has the potential to enhance both student learning and program effectiveness, and AI can support these dual outcomes in multiple ways. This potential is grounded in a well‐established understanding that assessment must be purposefully designed, clearly aligned with program outcomes, and continuously used to drive improvement, all principles affirmed across the leadership education literature (Hastings & Rosch, 2025; Pernier et al., 2025). Assessment is, at its core, “the process of defining, selecting, designing, collecting, analyzing, interpreting, and using information to increase students' learning and development” (Erwin, 1991, as cited in Pernier et al., 2025, p. 100), and it encompasses a spectrum of purposes — from gauging individual student learning to evaluating overall program effectiveness and informing continuous quality improvement (DeSawal and Peck 2022).

The distinction matters: what we assess, how we assess it, and for whom we are generating evidence each shape the design decisions leadership educators must make (DeSawal and Peck 2022; Hastings 2022). That complexity is further compounded when leadership itself is understood as a collective, relational, and socially constructed process, as group‐level outcomes and dialogic processes may require fundamentally different assessment methodologies than those designed to capture individual competency growth (Martin et al. 2022).

For formative assessment, AI can guide student self‐assessment by asking adaptive questions based on previous responses, helping students identify their current capacities more accurately, creating personalized development plans based on competency profiles, and providing real‐time feedback on student work that would be impossible for human educators to deliver at scale (Piech et al. 2021; van der Kleij et al., 2023). The efficacy of AI‐enhanced feedback loops in educational contexts has been demonstrated empirically: Kestin et al. (2025) found in a randomized controlled trial that college students using a pedagogically grounded AI tutor learned significantly more in less time compared to peers in active learning classrooms and reported higher levels of engagement and motivation.

For summative assessment, AI can help document student growth by identifying patterns across multiple assessment points, support portfolio development by helping students curate and present their best evidence of learning, analyze competency demonstrations using natural language processing to identify which competencies students are exhibiting in their reflections or performance tasks, and recognize patterns across cohorts that inform program improvements. Mixed methods approaches — pairing AI‐assisted quantitative pattern recognition with qualitative inquiry — are particularly well suited to capturing leadership's complexity, as neither data source alone is sufficient to evaluate a program's full impact (Hastings 2022). Group‐level assessment, in particular, benefits from these complementary lenses, since the dialogic, relational, and socio‐material dimensions of collective leadership practice are not easily captured through individual self‐report instruments alone (Martin et al. 2022).

These AI‐enhanced approaches don't replace human judgment in high‐stakes assessment decisions; they augment human capacity to make more informed judgments based on richer evidence. However, their adoption must be accompanied by deliberate ethical safeguards. Leadership educators should remain alert to bias risks embedded in AI feedback systems, which may reflect and reinforce inequities in how student competency is perceived and evaluated across race, gender, and other social identities (Montenegro and Jankowski 2020). Student data privacy and institutional governance of AI systems must be clearly established before any assessment data is processed algorithmically; the collection and use of sensitive developmental data requires transparent policies that students can meaningfully understand and consent to. Equity of access is an equally pressing concern. If AI‐enhanced formative feedback is available only to students with reliable technology access or only within better‐resourced programs, it may widen rather than close developmental gaps across student populations (Spencer and Smedick 2025).

There is also a risk of over‐reliance on algorithmic evaluation: when pattern‐recognition tools are mistaken for interpretive judgment, leadership educators may abdicate the critical sense‐making that assessment requires. Pernier et al. (2025) warn that metrics without direction lack purpose, and the same is true of algorithmically generated data without educator interpretation. Certain decisions should remain explicitly outside the scope of AI delegation entirely, including determinations about leadership identity development, readiness for advanced leadership roles, assessments that carry academic or professional consequences, and any evaluation requiring nuanced understanding of a student's relational or ethical growth. As Guthrie and Jenkins (2018) note, it is imperative that leadership educators understand not only why assessment is vital to program sustainability, but also how to accurately assess their programs and services — a responsibility that cannot be fully outsourced to automated systems.

## 3 Recommendations—A Path Forward

**FIGURE 2:**
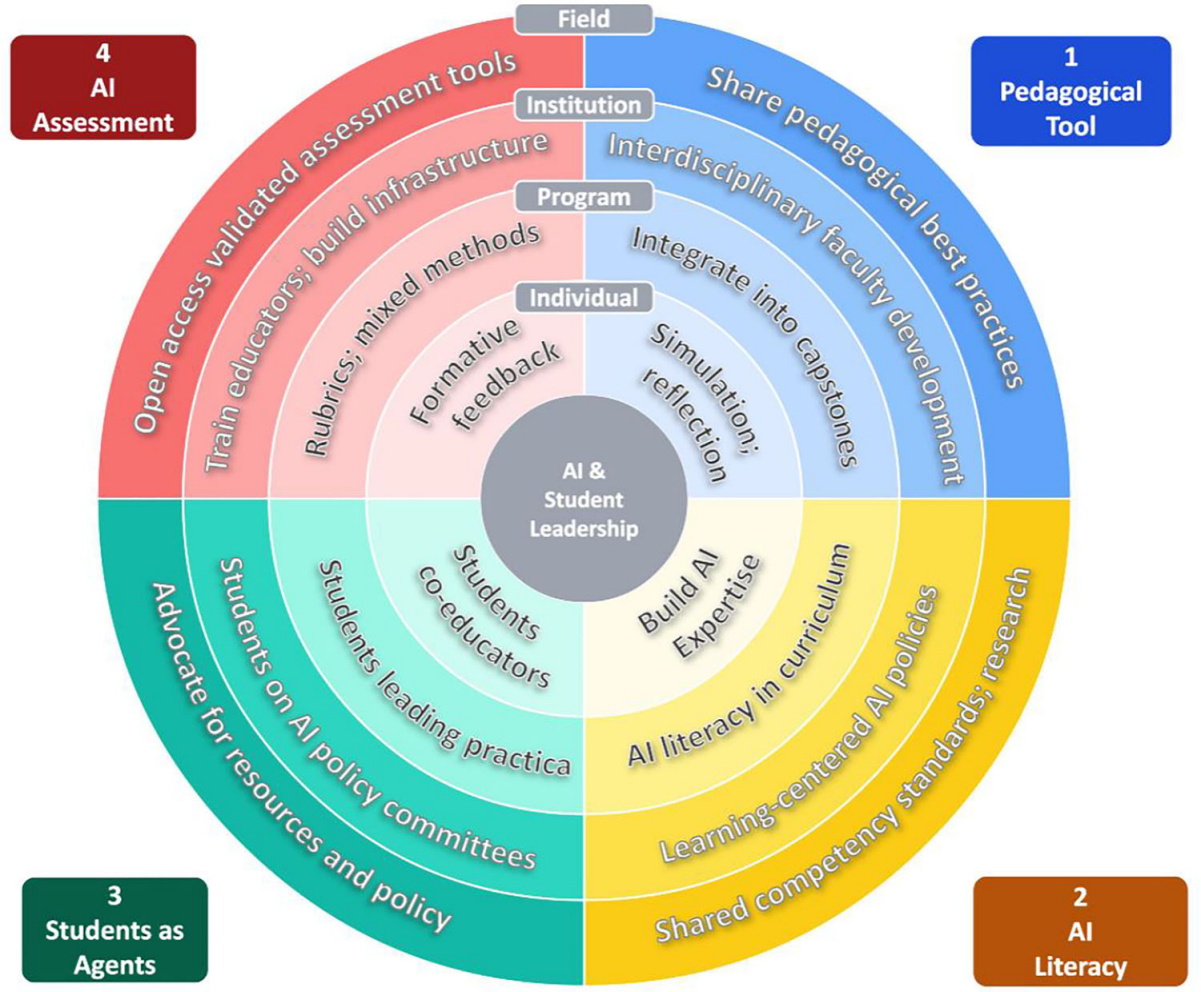
A Multi‐Level Framework for AI Integration in Student Leadership Development *Note*. Each concentric ring represents a level of action, innermost to outermost: Individual Educator → Program → Institution → Field. Each quadrant represents one of four imperatives. Reading outward shows how each imperative scales from personal practice to field‐wide change.

Translating these four imperatives into practice requires coordinated action across multiple levels of the leadership education ecosystem. Figure 2 maps this work visually, organizing implementation across four levels — individual educator, program, institution, and field — and illustrating how each imperative scales from personal pedagogical choices to collective, field‐wide change. The sections below elaborate the specific actions available at each level.

### 3.1 Level 1: Individual Educator Actions

Individual leadership educators can begin meaningful AI integration immediately through pedagogical creativity rather than institutional approval.

- **Integrate AI‐assisted reflection and dialogue**. Use AI tools to generate prompts for leadership reflection, provide initial feedback that students build upon with peers, or create personalized scenarios based on students' contexts. Model ethical AI use by demonstrating your decision‐making process—what you delegate to AI, what requires human judgment, and when you deliberately avoid AI.
- **Redesign assessment for AI integration**. Shift from plagiarism detection to learning enhancement. Ask students to use AI tools and critically evaluate outputs, explaining what they would retain or discard. Make AI use transparent rather than prohibited. Use AI to provide rapid initial feedback on low‐stakes assignments, freeing time for deeper engagement on high‐stakes work.
- **Build expertise through practice**. Engage in professional development to understand AI capabilities, limitations, and ethics. Join communities of practice. Recognize student expertise—position them as co‐educators who share experiences navigating AI tools and ethical questions.
- **Develop AI leadership scenarios**. Create case discussions addressing realistic dilemmas: adopting tools that might reduce headcount, ensuring systems don't perpetuate discrimination, determining when AI‐generated content is appropriate.

### 3.2 Level 2: Programmatic Redesign

Leadership development programs must systematically integrate AI across curricula and co‐curricular experiences through coordinated redesign.

- **Audit and revise leadership competency frameworks**. Identify gaps in AI literacy within existing frameworks. Embed AI concepts throughout curricula and/or programming rather than adding separate modules. Students should encounter AI repeatedly with increasing sophistication.
- **Redesign leadership development signature experiences**. Integrate AI into capstone projects, internships, and leadership practicums. Students might lead organizational AI policy development, assess AI implementation ethics, or design AI‐enhanced leadership initiatives.
- **Transform leadership assessment approaches**. Move beyond traditional surveys and written reflection assignments to multimodal assessments: AI‐enhanced presentations, ethical analysis of AI case studies, or portfolios demonstrating thoughtful AI use. Develop rubrics that explicitly evaluate AI literacy alongside traditional leadership competencies.
- **Establish peer learning structures**. Create communities where educators share AI integration strategies, troubleshoot challenges, and develop collective expertise.

### 3.3 Level 3: Institutional Transformation

Institutions must create infrastructure supporting AI integration while maintaining pedagogical values and addressing ethical concerns.

- **Develop AI policies centering learning**. Establish institution‐wide guidelines for ethical AI use in education. Policies should enable innovation while preventing harm, avoiding both blanket prohibitions and uncritical adoption.
- **Position students as institutional partners**. Include students in AI policy development committees, implementation teams, and ethical review processes. This provides authentic leadership opportunities while ensuring policies remain grounded in student realities.
- **Invest in faculty and staff development infrastructure**. Provide ongoing workshops, communities of practice, and one‐on‐one consultation supporting educators in AI integration. Create incentives for innovative AI pedagogy.
- **Build assessment capacity**. Train educators in designing AI‐integrated assessments and evaluating AI literacy. Provide technological infrastructure enabling sophisticated assessment approaches.
- **Foster interdisciplinary collaboration**. Connect leadership educators with computer science faculty, ethicists, and social scientists studying AI implications. Cross‐disciplinary partnerships enrich both leadership education and AI scholarship.

### 3.4 Level 4: Field‐Level Initiatives

Professional associations and the broader leadership education community must advance collective understanding and establish shared standards.

- **Establish communities of practice**. Create networks for sharing promising practices, research findings, and lessons learned. Professional associations should facilitate these connections through conferences, publications, and online platforms.
- **Develop shared assessment tools**. The field needs validated instruments measuring AI literacy as a leadership competency. Collaborative development ensures tools are rigorous, practical, and widely applicable.
- **Conduct research on effectiveness**. Systematically study how AI integration affects leadership learning outcomes, skill development, and student preparation. Share findings broadly to inform evidence‐based practice.
- **Establish shared AI literacy competency standards**. Professional associations and accrediting bodies should collaborate to define what AI literacy means as a leadership competency across institutional contexts, providing programs with shared language, benchmarks, and developmental progressions that enable consistent integration without mandating uniformity.
- **Advocate for resources and policy changes**. Professional associations should articulate resource needs for effective AI integration and advocate with policymakers, administrators, and funders.

## 4 Conclusion

The field of leadership development faces a critical choice: proactively engage with AI transformation or passively allow AI to reshape higher education without leadership educators' voice and expertise. These imperatives—AI as pedagogical tool, AI literacy as leadership competency, student agency in transformation, and AI‐enhanced assessment—provide a framework for meaningful engagement.

The path forward requires coordinated action across all levels. Individual educators must experiment with AI integration, programs must redesign curricula systematically, institutions must establish supportive infrastructure, and the field must build collective knowledge. Each level reinforces the others: educator innovations inform programmatic redesign, programmatic changes shape institutional policies, and field‐level initiatives support local implementation.

This engagement is both opportunity and responsibility. By modeling thoughtful AI integration, leadership education can demonstrate to higher education how to leverage powerful tools while maintaining pedagogical integrity, fostering critical thinking, and centering human connection. Simultaneously, leadership educators prepare students for leadership challenges they will face. This includes helping students develop capacities to make wise decisions about AI implementation, think critically about AI's societal implications, and maintain human judgment amid technological change.

The imperatives outlined here are ambitious but achievable. They require neither comprehensive AI expertise nor massive resource investment; they require commitment to thoughtful engagement and willingness to learn alongside students. Leadership educators have always prepared students to navigate ambiguity, make ethical decisions under uncertainty, and create positive change in complex environments. Engaging with AI meaningfully is simply extending this core mission into a new domain.

This vision is echoed in Yang and Jiménez‐Luque's (2025) Leadership Tree Model, which integrates AI‐enhanced learning, critical leadership perspectives, and the Leadership Learning Framework into a unified developmental framework — grounding technological adaptability in ethical engagement and positioning leadership education as a continuous, regenerative process rather than a fixed skill acquisition path. The question is not whether leadership education will grapple with AI, but whether we will do so intentionally and proactively, positioning ourselves and our students as agents of thoughtful transformation rather than passive recipients of technological change.
